# A retrospective analysis of treatment patterns, overall survival, and real-world disease-free survival in early-stage non-small cell lung cancer following complete resection

**DOI:** 10.1186/s12890-024-03138-y

**Published:** 2024-07-10

**Authors:** Xiaohan Hu, Diana Chirovsky, Mark S. Walker, Yuexi Wang, Alpana Kaushiva, Jon Tepsick, Ayman Samkari

**Affiliations:** 1 Merck & Co., Inc., P.O. Box 2000, 126 East Lincoln Avenue, Rahway, NJ 07065 USA; 2 ConcertAI, LLC, 1120 Massachusetts Ave., Cambridge, MA 02138 USA

**Keywords:** Real-world clinical outcomes, Early-stage NSCLC, Complete resection, Recurrence, Adjuvant chemotherapy

## Abstract

**Background:**

Real-world data regarding patient characteristics, adjuvant treatment patterns, and long-term survival outcomes are needed to better understand unmet needs among patients with completely resected early-stage non-small cell lung cancer (NSCLC).

**Methods:**

Electronic medical records from the U.S.-based ConcertAI Patient360™ database were analyzed in patients with stage IB-IIIA NSCLC who underwent complete resection prior to March 1, 2016. Patients were followed until death or July 1, 2021. This study evaluated adjuvant chemotherapy use, and overall survival (OS) and real-world disease-free survival (rwDFS) outcomes using the Kaplan–Meier method. The correlation between OS and rwDFS was assessed using the Kendall rank test. Among patients who did not recur 5 years following surgery, landmark analyses of OS and rwDFS were conducted to understand the subsequent survival impact of remaining disease-free for at least 5 years.

**Results:**

Data from 441 patients with completely resected stage IB-IIIA NSCLC were included. About 35% of patients received adjuvant chemotherapy post-resection. Median OS and rwDFS from resection were 83.1 months and 42.4 months, respectively. The 5-year OS and rwDFS rates were 65.7% and 42.1%, respectively. OS and rwDFS were positively correlated (Kendall rank correlation coefficient = 0.67; *p* < 0.0001). Among patients without recurrence within 5 years after resection, the subsequent 5-year OS and rwDFS survival rates were 52.9% and 36.6%, respectively.

**Conclusions:**

Use of adjuvant chemotherapy was low, and the overall 5-year OS rate remained low despite all patients having undergone complete resection. Patients who remained non-recurrent over time had favorable subsequent long-term survival.

**Supplementary Information:**

The online version contains supplementary material available at 10.1186/s12890-024-03138-y.

## Background

Approximately 85% of all lung cancer cases are non-small cell lung cancer (NSCLC) [1], and 20–30% of these are diagnosed with localized or regional early-stage disease [2, 3]. The overall survival (OS) of these patients varies depending on stage of disease. The rate of 5-year OS after resection for patients with early-stage NSCLC has been shown to range from a high of 82% for stage IA to a low of 36% for stage IIIA [4].

Although not all patients who are eligible for resection receive it, the NCCN Clinical Practice Guidelines in Oncology (NCCN Guidelines®) recommend that patients with stage IB to IIIA disease with N0 or N1 nodes undergo surgical resection [5]. The primary factors that determine eligibility for adjuvant chemotherapy include stage of disease, pre-existing comorbidities [6], and whether the patient had a pathologic complete response after complete resection [7, 8]. The NCCN Guidelines® recommend four cycles of adjuvant chemotherapy for patients with stage IB and IIA disease who are considered high-risk, such as those as with poorly differentiated tumors, vascular invasion, tumors > 4 cm, wedge resection, unknown lymph node status, or visceral pleural involvement; and for all patients with American Joint Committee on Cancer (AJCC) 8^th^ edition stage IIB-IIIA disease after complete resection [5]. Cisplatin-containing doublet regimens (with pemetrexed, gemcitabine, docetaxel, vinorelbine, or etoposide) have been recommended as a treatment option by the European Society of Medical Oncology [6] and in the NCCN Guidelines [5]. However, previous research has shown that adherence to these guidelines has been limited in the U.S. A recent observational study of 2,833 patients in the U.S. with early-stage NSCLC showed that only 57% received any adjuvant chemotherapy post-resection, and only 44% received the minimum recommended four cycles, even though adjuvant chemotherapy was indicated for all patients in the study [9].

This underuse of adjuvant chemotherapy may be attributable to perceptions of treatment effectiveness. Cisplatin-based adjuvant chemotherapy has been shown to have modest efficacy in improving survival outcomes [10, 11]. Even with adjuvant chemotherapy, 30–50% of patients with completely resected early-stage NSCLC ultimately experience a recurrence [12, 13], which is then associated with a significant negative impact on OS [14, 15]. Most previous studies have had limited follow-up with which to evaluate long-term outcomes, and few studies have investigated adjuvant chemotherapy use specifically in patients with completely resected early-stage disease, regardless of risk status, using more recent data [9, 16]. Additional real-world data, defined as “data relating to patient health status and/or delivery of health care routinely collected” from sources that include electronic medical records (EMRs) and claims [17], are therefore needed. These real-world data should include patient characteristics, treatment use in the adjuvant setting, and long-term survival outcomes to better understand the unmet needs of this population. Such real-world data should include methods like human document review to provide information about outcomes that is complete and accurate.

OS is the gold standard for evaluating the efficacy of new oncologic therapies. However, it can take many years for OS data to mature, especially in patients with early-stage disease. Disease-free survival (DFS) has been accepted by regulatory agencies in evaluation of new oncologic therapies, and evidence based on clinical trial data has demonstrated very good correlation of DFS and OS in patients with completely resected early-stage NSCLC [18–20]. The hypothesis of this study was that findings from a cohort of patients receiving care in real-world practice would demonstrate a positive, significant correlation between rwDFS and OS. Examination of these outcomes and of the correlation of DFS and OS in real-world data would complement the existing clinical trial evidence.

Here, we report the rate of adjuvant treatment use, real-world adjuvant treatment patterns, and the correlation between real-world DFS (rwDFS) and OS among patients with stage IB-IIIA NSCLC who underwent complete resection. This study also reports the long-term survival outcomes for patients and includes additional analysis among patients who did not experience a recurrence by certain landmarks, to delve deeper into the experience of these patients. Findings from direct comparison of outcomes between patients who experienced a recurrence versus those who did not are reported in a separate manuscript and published elsewhere [21].

## Methods

### Study design and patients

This was a retrospective, non-interventional cohort study using EMR data from the ConcertAI Patient360™ database. The database includes de-identified structured and unstructured data elements (text and image documents, e.g., physician progress notes). The unstructured information is curated by experienced Clinical Research Nurses (CRNs) and linked to the structured field data for analysis. Data were obtained from a geographically diverse set of primarily community oncology practices (80–90%), drawn from both rural and urban centers within the U.S. The data used in this study were all collected originally for clinical use in the practice setting and are therefore considered secondary data. Only de-identified data were utilized in the analysis, and unique patient identifiers were completely removed from the analytical dataset. This research project was reviewed by an institutional review board (IRB; Advarra, Columbia, MD, U.S.) and found to be exempt from IRB oversight.

Patients were eligible for inclusion if they had a provider-documented primary diagnosis of stage IB-IIIA NSCLC and provider-documented residual tumor stage of R0 post-resection (complete resection). All patients underwent complete resection prior to March 1, 2016 and were followed until death or July 1, 2021, whichever occurred first, to allow for a minimum 5-year theoretical follow-up. Manual chart review of EMR data, including diagnosis, staging, complete resection, and disease recurrence, was conducted by trained CRNs, who participated in study-specific case report form training to minimize potential biases in chart interpretation. All data curated from the ConcertAI Patient360™ database by CRNs underwent an independent quality control review, with evaluation for consistency, completeness, and outlier values. Patients were excluded from the study if they received neoadjuvant chemotherapy or radiotherapy, had a record of participation in an interventional clinical trial at any time prior to recurrence, or had an Eastern Cooperative Oncology Group (ECOG) performance status of 2 or above. If ECOG status was not documented, manual review by CRNs was conducted to determine if there was any indication of impaired performance present in the unstructured data, and was used in lieu of an observed ECOG indication.

### Study variables

Patient demographic and clinical characteristics at the time of initial diagnosis were assessed descriptively, and included age, gender, race, region, comorbidities, stage, histology, and performance status. The date and type of surgery at complete resection were also captured. Adjuvant systemic anticancer treatment patterns were assessed, including evaluation of the distribution and duration of treatment regimens. Combination regimens in this study were defined as multiple treatments initiated within the same 28-day period, with no agent being discontinued or held for more than 120 days. A change of regimen was defined as the use of any new agent that was added more than 28 days after the initiation of a regimen, or discontinuation of an agent more than 120 days before other agents in the regimen were stopped. Reintroduction of a regimen more than 120 days after its previous discontinuation was also considered a change of regimen. The substitution of cisplatin for carboplatin (and vice versa), and the substitution of paclitaxel for albumin-bound paclitaxel or docetaxel (and vice versa) were not considered a new regimen.

OS was defined as the interval from the date of complete resection until death due to any cause. Patients were censored at the date of their last medical record if there was no evidence of death. Date of death was ascertained from the patient’s clinical records, from Social Security Death Index records, or from associated obituary records linked to the ConcertAI Oncology Dataset. rwDFS was defined as the interval from the date of complete resection to the date of the first recurrence event (locoregional recurrence or distant recurrence, based on provider documentation and confirmed via manual chart review), new diagnosis of other primary cancer other than non-melanoma skin cancer, or date of death, whichever occurred first. Patients were censored at the date of their last medical record if there was no evidence of recurrence or death.

### Statistical analyses

Descriptive statistics were used to summarize patient characteristics and to evaluate treatment patterns in the adjuvant setting. All analyses were conducted using SAS version 9.4 (SAS Institute Inc., Cary, NC, U.S.).

Kaplan–Meier analyses were conducted to examine OS and rwDFS, both measured from the date of complete resection, overall and by stage at initial diagnosis. The percentage of patients surviving to 1, 3, and 5 years after resection was examined for both OS and rwDFS, overall and separately for patients in each stage stratum. The Kendall rank correlation coefficient was used to examine the correlation between OS and rwDFS from complete resection in this study population. There were no prespecified thresholds for considering the utility of rwDFS as a proxy measure for OS. However, both a 95% confidence interval around the coefficient estimate, as well as an associated *p*-value, were calculated using large sample approximations. To better understand how long patients would continue to survive and/or remain disease-free after having remained disease-free for a specified interval, OS and rwDFS from defined landmark timepoints were assessed among patients without a recurrence or death by those landmark points. The landmark analysis used Kaplan–Meier methods, and the landmark timepoints were defined as 1, 3, and 5 years after complete resection. Landmark analysis was used to manage immortal time bias that would otherwise result due to recurrence occurring at different time points.

## Results

### Patient characteristics

A total of 441 patients were included in this study after applying all eligibility criteria (see Supplementary Figure 1). Of these, 153 (34.7%) were diagnosed with stage IB disease at time of initial diagnosis, 183 (41.5%) were diagnosed with stage II disease, and 105 (23.8%) were diagnosed with stage IIIA disease (Table 1). The median age at initial diagnosis was 67 years, and about half of the patients were male. Most patients were White (83.9%). Additionally, most patients were from either the Midwest or Southern regions of the U.S. (41.3% and 34.7%, respectively). The most common disease histology observed at initial diagnosis was adenocarcinoma not otherwise specified (NOS) (50.7%), followed by squamous cell carcinoma NOS (26.6%), and adenocarcinoma with mixed subtypes (7.2%).

**Table 1:** Patient characteristics at initial diagnosis for patients with completely resected stage IB-IIIA NSCLC, overall and by stage

Variable/Statistic	Overall (*N* = 441)	Stage IB (*N* = 153)	Stage II (*N* = 183)	Stage IIIA (*N* = 105)
Age at initial diagnosis, median (Q1-Q3), years	67.0 (59.0, 73.0)	67.0 (60.0, 73.0)	67.0 (60.0, 75.0)	67.0 (58.0, 72.0)
Sex, Male, n (%)	222 (50.3%)	76 (49.7%)	96 (52.5%)	50 (47.6%)
Race, White, n (%)	370 (83.9%)	126 (82.4%)	152 (83.1%)	92 (87.6%)
U.S. Region, n (%)				
Midwest	182 (41.3%)	70 (45.8%)	74 (40.4%)	38 (36.2%)
Northeast	53 (12.0%)	24 (15.7%)	16 (8.7%)	13 (12.4%)
South	153 (34.7%)	45 (29.4%)	70 (38.3%)	38 (36.2%)
West	48 (10.9%)	11 (7.2%)	21 (11.5%)	16 (15.2%)
Unknown/undocumented	5 (1.1%)	3 (2.0%)	2 (1.1%)	0 (0.0%)
Disease histology, n (%)^a^				
Adenocarcinoma with mixed subtypes	24 (7.2%)	8 (6.6%)	11 (8.3%)	5 (6.2%)
Adenocarcinoma, NOS	170 (50.7%)	58 (47.9%)	72 (54.1%)	40 (49.4%)
Squamous cell carcinoma, NOS	89 (26.6%)	34 (28.1%)	34 (25.6%)	21 (25.9%)
Other	52 (15.5%)	21 (17.4%)	16 (12.0%)	15 (18.5%)
Performance status, n (%)				
ECOG Score—0	75 (17.0%)	20 (13.1%)	35 (19.1%)	20 (19.0%)
ECOG Score—1	53 (12.0%)	14 (9.2%)	25 (13.7%)	14 (13.3%)
No indication of impaired performance	313 (71.0%)	119 (77.8%)	123 (67.2%)	71 (67.6%)
Comorbidities, n (%)^b^				
Cerebrovascular disease	22 (5.0%)	8 (5.2%)	10 (5.5%)	4 (3.8%)
Chronic obstructive pulmonary disease	114 (25.9%)	37 (24.2%)	42 (23.0%)	35 (33.3%)
Diabetes	52 (11.8%)	21 (13.7%)	21 (11.5%)	10 (9.5%)
Smoking status, n (%)				
Current	124 (28.1%)	51 (33.3%)	47 (25.7%)	26 (24.8%)
Past (Not current)	221 (50.1%)	71 (46.4%)	90 (49.2%)	60 (57.1%)
Never	70 (15.9%)	20 (13.1%)	35 (19.1%)	15 (14.3%)
Unknown	26 (5.9%)	11 (7.2%)	11 (6.0%)	4 (3.8%)
Type of surgery performed for complete resection, n (%)				
Lobectomy of lung	243 (55.1%)	86 (56.2%)	114 (62.3%)	43 (41.0%)
Thoracoscopic lobectomy of lung	100 (22.7%)	41 (26.8%)	37 (20.2%)	22 (21.0%)
Total pneumonectomy	23 (5.2%)		11 (6.0%)	12 (11.4%)
Thoracoscopic wedge resection of lung	18 (4.1%)	9 (5.9%)	5 (2.7%)	4 (3.8%)
Wedge resection	29 (6.6%)	10 (6.5%)	6 (3.3%)	13 (12.4%)
Other	28 (6.3%)	7 (4.6%)	10 (5.5%)	11 (10.5%)
Theoretical follow-up (in months)^c^				
Median	91.9	94.8	88.9	94.7
Q1, Q3	77.3, 114.8	78.0, 131.6	76.0, 110.2	79.4, 114.4
Min, Max	62.6, 256.3	62.6, 214.0	62.6, 210.9	62.6, 256.3

*AIDS* Acquired immune deficiency syndrome, *ECOG* Eastern Cooperative Oncology Group, *HIV* human immunodeficiency virus, *NOS* not otherwise specified, *NSCLC* non-small cell lung cancer, *Q* quartile, *U.S.* United States

^a^Among *n* = 335 (76%) patients with documented histology

^b^Comorbidities examined in this study include myocardial infarction, congestive heart failure, peripheral vascular disease, cerebrovascular disease, Alzheimer’s or other dementia, chronic obstructive pulmonary disease, connective tissue disease, ulcer disease, diabetes, renal disease, leukemia, lymphoma, cirrhosis or other serious liver disease, HIV + /AIDS, and autoimmune disease. Comorbid conditions with < 5% prevalence are not shown

^c^From complete resection to data cut-off

Seventeen percent of patients had an ECOG performance status score of 0 at initial diagnosis, 12% had a score of 1, and there was no indication of impaired performance for the remaining 71% of patients (Table 1). Half of the patients in this sample (50.1%) were documented as past smokers at the time of their initial diagnosis, and 28.1% were documented as current smokers. The most common type of surgery performed for complete resection was lobectomy of lung (55.1%) followed by thoracoscopic lobectomy of lung (22.7%). The most common comorbid conditions observed among patients included chronic obstructive pulmonary disease and diabetes (25.9% and 11.8%, respectively). Median observed follow-up time from complete resection to data cut-off was 91.9 months. Most patients (77.1%) had a complete resection within 10 years of the study end date (July 1, 2021), and the earliest date of complete resection was January 2020.

### Adjuvant systemic therapy treatment patterns

**Fig. 1:**
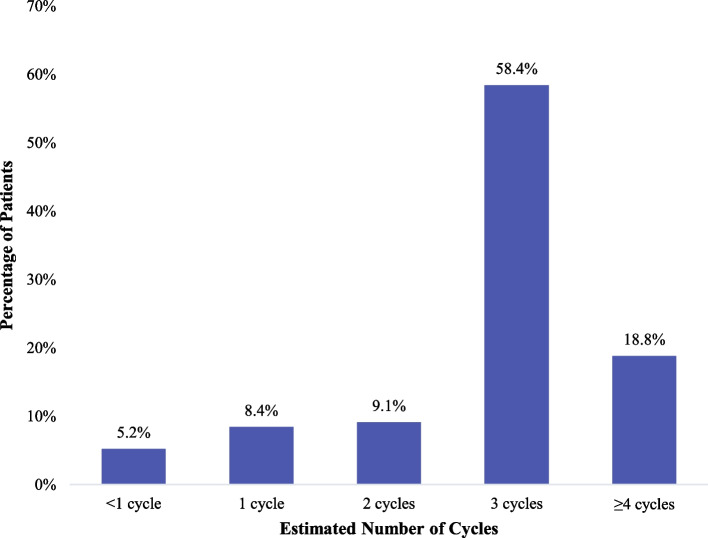
Duration of first adjuvant therapy for patients with completely resected stage IB-IIIA NSCLC who received adjuvant systemic therapy A cycle is estimated as a treatment duration of approximately 21 days

A total of 154 patients (34.9%) received adjuvant systemic therapy after complete resection with variation observed across disease stages: 50.5% of patients with stage IIIA disease, 42.1% with stage II disease, and 15.7% with stage IB disease received adjuvant systemic therapy, respectively (see Supplementary Table 1). Among patients who initiated any adjuvant systemic therapy, the majority received platinum-based chemotherapy (*N* = 136, 88.3%), including 90 (58.4%) who received cisplatin-based therapies, and 46 (29.9%) who received carboplatin-based chemotherapy, with the remaining patients receiving another type of therapy (*N* = 18, 11.7%) (Table 2). The median duration of the first adjuvant therapy regimen was 2.1 months (Q1-Q3: 2.1–2.3 months); 18.8% of patients received at least four cycles (measured as treatment duration ≥ 84 days) and 86.4% received at least two cycles (measured as treatment duration ≥ 42 days) (Fig. 1). Among the total sample of 154 patients who received a first adjuvant therapy, 26 received a second adjuvant therapy (16.8%), and 9 received a third adjuvant therapy (5.8%) (see Supplementary Table 1).

**Table 2:** Treatment distribution for the first adjuvant systemic therapy for patients with completely resected stage IB-IIIA NSCLC

Adjuvant therapy, first regimen, n (%)	Adjuvant therapy (*N* = 154)
**Platinum-based chemotherapy**	136 (88.3%)
**Carboplatin-based regimen**	**46 (29.9%)**
Carboplatin, paclitaxel	19 (12.3%)
Carboplatin, pemetrexed	13 (8.4%)
Carboplatin, docetaxel	8 (5.2%)
Carboplatin, etoposide	4 (2.6%)
Carboplatin, paclitaxel, pemetrexed	1 (0.6%)
Bevacizumab, carboplatin, paclitaxel	1 (0.6%)
**Cisplatin-based regimen**	**90 (58.4%)**
Cisplatin, docetaxel	36 (23.4%)
Cisplatin, pemetrexed	32 (20.8%)
Cisplatin, vinorelbine	13 (8.4%)
Cisplatin, etoposide	4 (2.6%)
Cisplatin, gemcitabine	3 (1.9%)
Cisplatin, paclitaxel	1 (0.6%)
Bevacizumab, cisplatin, docetaxel	1 (0.6%)
**Other systemic therapy**	**18 (11.7%)**
Targeted therapy^a^	9 (5.8%)
Other chemotherapy^b^	6 (3.9%)
Immunotherapy^c^	3 (1.9%)

^a^The following targeted therapies were used: erlotinib *n* = 6; crizotinib *n* = 1; dabrafenib, trametinib *n* = 1; sorafenib *n* = 1

^b^The following other chemotherapies were used: cyclophosphamide, doxorubicin, vincristine *n* = 1; paclitaxel, trastuzumab *n* = 1; gemcitabine *n* = 1; methotrexate *n* = 1; pemetrexed *n* = 1; temozolomide *n* = 1

^c^The following immunotherapies were used: pembrolizumab *n* = 2; durvalumab *n* = 1

### Overall survival and real-world disease-free survival

**Fig. 2:**
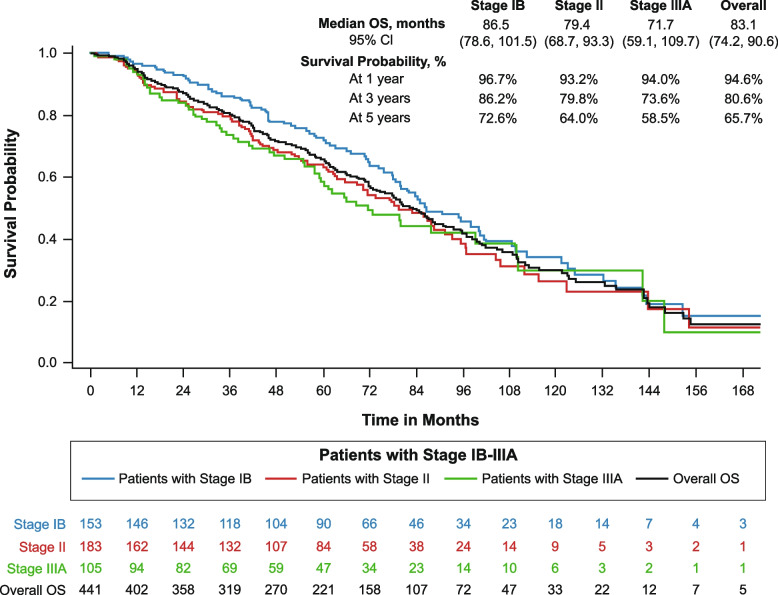
Kaplan–Meier analysis of overall survival from complete resection for patients with stage IB-IIIA OS, overall survival

Median OS in the overall sample was 83.1 months (*n* = 441; 95% CI: 74.2, 90.6) and tended to decrease with increased disease stage (Fig. 2); median OS was 86.5 months in patients with stage IB NSCLC (*n* = 153; 95% CI: 78.6, 101.5), 79.4 months in patients with stage II NSCLC (*n* = 183; 95% CI: 68.7, 93.3), and 71.7 months in patients with stage IIIA NSCLC (*n* = 105; 95% CI: 59.1, 109.7). The 5-year survival probability for the overall sample was 65.7% (95% CI: 60.7%, 70.1%), and also tended to decrease with increased disease stage: 72.6% for patients with stage IB disease, 64.0% for those with stage II disease, and 58.5% for those with stage IIIA disease.

**Fig. 3:**
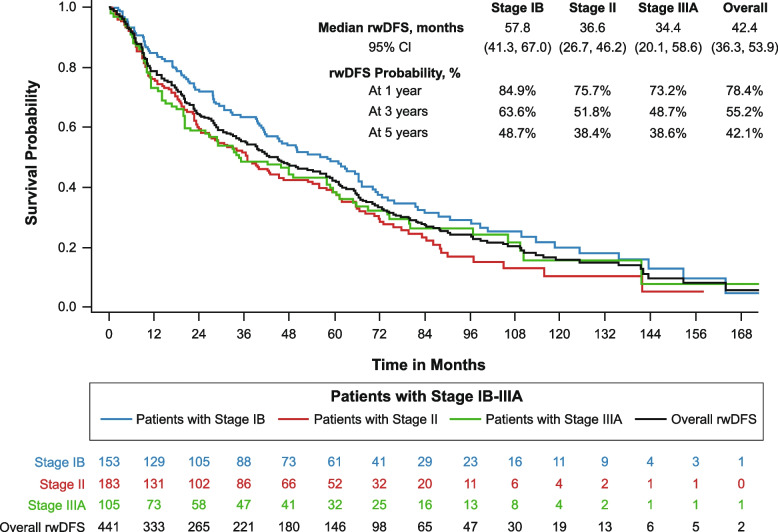
Kaplan–Meier analysis of real-world disease-free survival from complete resection for patients with stage IB-IIIA rwDFS, real-world disease-free survival

Median rwDFS in the overall sample was 42.4 months (*n* = 441; 95% CI: 36.3, 53.9) and tended to decrease with increased disease stage (Fig. 3). Median rwDFS was 57.8 months among patients with stage IB NSCLC (*n* = 153; 95% CI: 41.3, 67.0), 36.6 months in those with stage II NSCLC (*n* = 183; 95% CI: 26.7, 46.2), and 34.4 months in those with IIIA disease (*n* = 105; 95% CI: 20.1, 58.6). The 5-year rwDFS probability for the overall sample was 42.1% (95% CI: 37.2%, 46.9%). The 5-year rwDFS probabilities for each stage group reflect the pattern of the rwDFS medians, at 48.7% for patients with stage IB NSCLC, 38.4% for those with stage II NSCLC, and 38.6% for those with stage IIIA NSCLC. The Kendall’s Tau Correlation Coefficient estimate was 0.67 (95% CI: 0.62, 0.72, *p* < 0.0001), indicating that OS has a significant, moderately positive correlation with rwDFS after complete resection in this cohort.

### Landmark analysis of overall survival and real-world disease-free survival among patients without recurrence by 1-, 3- and 5-years following surgery

For patients who remained disease-free by the 1-year (*n* = 333), 3-year (*n* = 221), and 5-year (*n* = 145) landmarks after complete resection, the median OS from the respective landmarks was 83.5 months (95% CI: 73.9, 92.4), 75.6 months (95% CI: 64.5, 96.3), and 64.7 months (95% CI: 49.6, 83.1) (Table 3; see Supplementary Figure 2). The probabilities that the patients would remain alive at 5 years after each landmark, i.e., at 6, 8, and 10 years following complete resection, were 65.2%, 63.7%, and 52.9%. For patients who were recurrence free for at least 5 years, the median OS for patients with stage IB, II, and IIIA disease was 72.3 months, 55.4 months, and 82.3 months, respectively (*p* = 0.971).

**Table 3:** Landmark analyses of rwDFS/OS among patients without recurrence by each landmark timepoint

	**1 year following surgery**	**3 years following surgery**	**5 years following surgery**
No. of patients	333	221	145
No. of OS events	161	88	53
No. of rwDFS events^a^	219	123	73
Median OS (95% CI) in months	83.5 (73.9, 92.4)	75.6 (64.5, 96.3)	64.7 (49.6, 83.1)
Median rwDFS (95% CI) in months	51.1 (45.8, 56.6)	47.1 (38.0, 60.5)	44.4 (28.1, 55.4)
OS probability, %			
At 1 year after each landmark	96.00%	95.90%	88.10%
At 3 years after each landmark	83.00%	78.80%	73.80%
At 5 years after each landmark	65.20%	63.70%	52.90%
rwDFS probability, %			
At 1 year after each landmark	81.40%	85.30%	77.90%
At 3 years after each landmark	60.10%	59.40%	56.90%
At 5 years after each landmark	41.80%	43.40%	36.60%

OS was defined as the interval from the date of complete resection until death due to any cause

rwDFS was defined as the interval from the date of complete resection to the date of the first recurrence event (locoregional recurrence or distant recurrence, based on provider documentation), new diagnosis of other primary cancer other than non-melanoma skin cancer, or date of death, whichever occurred first

*CI* confidence interval, *OS* overall survival, *rwDFS* real-world disease-free survival

^a^One patient had a new primary cancer as a recurrence event (multiple myeloma)

The median rwDFS from the respective landmarks after complete resection was 51.1 months (95% CI: 45.8, 56.6), 47.1 months (95% CI: 38.0, 60.5), and 44.4 months (95% CI: 28.1, 55.4) (Table 3; see Supplementary Figure 3). The probabilities that the patients would remain disease-free and alive at 5 years after each landmark, i.e., at 6, 8, and 10 years following complete resection, were 41.8%, 43.4%, and 36.6%, respectively. For patients who were recurrence free for at least 5 years, the median rwDFS for patients with stage IB, II, and IIIA disease was 49.4 months, 28.0 months, and 49.1 months, respectively (*p* = 0.339).

As events in a Kaplan–Meier analysis of rwDFS can be of two types (disease recurrence vs. death without an observed recurrence), we further analyzed the distribution of event type to better examine the effect of remaining disease-free over time on type of subsequent recurrence. Among patients without recurrence at 1 year after complete resection, 71.7% of subsequent rwDFS events were disease recurrence (see Supplementary Table 2). However, the corresponding rates of subsequent recurrence among patients who were non-recurrent at the 3-year and 5-year landmarks were 62.6% and 53.4%, respectively, suggesting that the longer a patient remained non-recurrent, the more likely it was that any subsequent rwDFS event was a death without evidence of recurrence.

## Discussion

This study examined adjuvant systemic therapy treatment patterns and survival outcomes among a real-world cohort of patients with completely resected early-stage NSCLC, drawn from a large database of primarily community oncology practices across the U.S. The findings showed that only approximately a third of patients receive adjuvant systemic therapy delivery, and that patients still experience poor survival outcomes, with a 5-year OS rate of approximately 65.7%. A moderate, significantly positive correlation was also demonstrated between OS and rwDFS.

This study adds to the literature by examining effectiveness outcomes with longer follow-up than has typically been observed in real-world populations of patients with early-stage NSCLC. All patients were required to have a minimum of 5 years of available theoretical follow-up, and the observed median follow-up was greater than 6 years. Additionally, this study sought to directly examine the impact of not having a recurrence through landmark analyses, and to our knowledge, is the only study to date that has adopted this approach. This approach was utilized to control immortal time bias that would otherwise be introduced by the timing of recurrence after complete resection. The findings from this study highlight the need for more effective adjuvant treatment options for patients with early-stage NSCLC who receive complete resection, and illustrate the outcomes among patients whose disease has not recurred over time.

There were several noteworthy findings from this study. A key observation was the underutilization of adjuvant chemotherapy. Overall, about 35% of patients in our study initiated adjuvant chemotherapy following primary surgery. This rate is lower than the 57% reported by Kehl and colleagues [9]. However, their study included patients being considered for a clinical trial, which may have enriched for patients more likely to receive guideline-concordant care and, it required that patients be indicated to receive adjuvant chemotherapy. Our study did not require this, and we did not have access to certain variables that determined the indication. Conversely, the rate of adjuvant chemotherapy use in our study was somewhat higher than what was reported by Williams and colleagues [16], but this may be attributable to our more recent data (2000–2021 in this study vs. 2001–2008 in Williams and colleagues).

We could not determine which study patients with stage I and II NSCLC were indicated to receive adjuvant chemotherapy, in part because we did not have some of the required risk factor information. However, all study-eligible patients with stage IIIA disease and no contraindications for adjuvant chemotherapy should have received it, since patients with presurgical chemotherapy exposure were excluded, but only half of these patients received it. Beyond this, the study showed that only about 19% of patients who received adjuvant chemotherapy received it for the recommended duration of at least four treatment cycles (measured as treatment duration ≥ 84 days). Together with the low rate at which patients received adjuvant treatment, our data clearly illustrate underutilization.

The study findings also illustrate the overall burden of this disease. We observed a median OS of only 83.1 months and a 5-year OS rate of just 65.7%, despite this being a population with completely resected early-stage NSCLC. Similarly, the median rwDFS was 42.4 months, and the estimated 5-year rwDFS rate was just 42.1%. We also found that patients with more advanced stage of disease at diagnosis had poorer outcomes, as is consistent with previous findings [4]. Newer perioperative treatments are emerging which may reduce the overall disease burden for patients with early-stage NSCLC [22–25]. The efficacy benefits from these pivotal clinical trials demonstrate the promise of newer therapies for patients with early-stage NSCLC, and the low rates of adjuvant chemotherapy also highlight the limitations of historical approaches involving the use of adjuvant chemotherapy alone.

This study evaluated the outlook for survival among patients who remained disease-free for defined intervals following complete resection. We examined these subpopulations to better understand the potential benefit of new therapies that may reduce or delay recurrence. The landmark analysis of OS shows that patients without a recurrence at the 1-year landmark after surgery had a 5-year survival probability after the landmark (6 years after surgery) of 65.2%. For patients without a recurrence at the 5-year landmark, the 5-year survival probability after the landmark (10 years after surgery) was 52.9%. We also evaluated the benefit of non-recurrence on subsequent rwDFS rates. The benefit of non-recurrence is suggested in the declining rate at which subsequent rwDFS events represent a disease recurrence, rather than a death without documentation of disease recurrence. Deaths without evidence of recurrence may represent deaths not directly attributable to lung cancer. The pattern suggests a decreasing likelihood that patients whose disease has not recurred over time will experience a subsequent recurrence. However, further research should investigate whether this trend is observed with evaluation of lung cancer-specific mortality in patients whose disease has not recurred over time.

Finally, as hypothesized, our study showed that rwDFS had a significant positive correlation with OS in this patient cohort. Based on previously established statistical thresholds, correlation coefficients ranging from 0.6 to 0.7 constitute a moderate relationship [26, 27]. As such, the observed correlation coefficient of 0.67 in this study represents a moderately strong relationship of rwDFS with OS. Although the association of OS and DFS has been examined previously in clinical trials, this study is the first we are aware of that has evaluated this relationship in the real-world setting for patients with early-stage NSCLC. We do not consider the evidence from this study alone as sufficient to support the use of DFS as a surrogate for OS. Rather, it should be viewed as complementary to existing clinical trial evidence, further supporting the use of DFS as a proxy measure for OS.

This study had several strengths. The database used in this study included patients who were selected from a geographically diverse area in the U.S. Study patients were sampled irrespective of registry participation, insurance program enrollment, or healthcare system membership, and hence should be reflective of treatment patterns and outcomes observed in the real-world population. The study also included data curated through manual review of unstructured EMR data, which provided more accurate and complete information compared to structured EMR data alone. Case eligibility regarding diagnosis, staging, and confirmation of complete resection was verified through manual review of EMR data by trained CRNs who had access to the entirety of the unstructured data, including pathology reports, progress notes, radiological scans, etc. Other variables, including performance status, histology, tumor response, and recurrence status were also manually curated, which establishes the greatest level of quality and completeness possible using retrospective real-world data, and allows for increased confidence in the study findings. Additionally, this study was innovative in its use of landmark analysis to control for immortal time bias. The study also examined outcomes from several different landmarks in an effort to evaluate the sensitivity of the findings to the selection of the landmark. Finally, the study also utilized a minimum theoretical follow-up period of at least 5 years since primary surgery, to allow for more comprehensive assessment of long-term survival outcomes.

Our study also had distinct limitations. First, the retrospective nature of this study lends itself to limitations such as data missingness and potential misclassification, and findings should be interpreted with these in mind. However, to minimize the impact of these limitations, the study employed manual review by highly trained CRNs to examine EMR data from structured and unstructured sources. Next, the study was conducted mainly with patients treated within community oncology practices in the U.S. Because of this, the findings may not be generalizable to patients treated in academic centers or in practices outside of the U.S. Additionally, the study did not include certain variables that are related to high-risk status in patients with stage IB and IIA NSCLC and that are necessary for exact mapping of staging of disease from the AJCC 6^th^ edition to the 7^th^ edition, such as tumor size. Finally, because the study required long-term follow-up of patients who had undergone complete resection prior to March 1, 2016, there was a limited number of patients in this study who had received immunotherapy and targeted therapy as front-line treatments. As a result, the findings from this study may not be generalizable to the outcomes conferred by more recently approved therapies.

## Conclusions

In conclusion, the results from this study align with prior research in showing underutilization of adjuvant chemotherapy in patients with completely resected early-stage NSCLC. The study also showed the substantial disease burden in this population as demonstrated by the observed overall 5-year OS and rwDFS rates. Additionally, the study provides evidence which complements previous findings for the use of DFS as a proxy measure for OS in this patient population. Future studies should continue to evaluate trends regarding compliance with guideline recommendations, especially considering the availability of newer treatments.

### Supplementary Information

Supplementary Material 1.

## Data Availability

The data that support the findings of this study are available from ConcertAI, LLC but restrictions apply to the availability of these data, which were used under license for the current study, and so are not publicly available. Data are however available from ConcertAI, LLC upon reasonable request (https://www.concertai.com/contact-us/).

## Abbreviations

AJCC: American Joint Committee on Cancer
CI: Confidence interval
CRN: Clinical Research Nurse
DFS: Disease-free survival
ECOG: Eastern Cooperative Oncology Group
EMR: Electronic medical record
IRB: Institutional review board
NCCN: National Comprehensive Cancer Network
NOS: Not otherwise specified
NSCLC: Non-small cell lung cancer
OS: Overall survival
rw: Real-world
U.S.: United States
