# Supplementary material for: A retrospective analysis of treatment patterns, overall survival, and real-world disease-free survival in early-stage non-small cell lung cancer following complete resection
Source: BMC Pulm Med. 2024 Jul 10;24:332. doi: 10.1186/s12890-024-03138-y (PMC11234548; doi:10.1186/s12890-024-03138-y)
Supplement: Supplementary file 1 — Supplementary Material 1. [file 12890_2024_3138_MOESM1_ESM.docx]

**Article title:** A Retrospective Analysis of Treatment Patterns, Overall Survival, and Real-World Disease-Free Survival in Early-Stage Non-Small Cell Lung Cancer Following Complete Resection

**Journal name:** BMC Pulmonary Medicine

**Author names:** Xiaohan Hu*, Diana Chirovsky, Mark S. Walker, Yuexi Wang, Alpana Kaushiva, Jon Tepsick, Ayman Samkari

***Affiliation and e-mail address of the corresponding author:** Xiaohan Hu, PhD, MPH, Merck & Co, Inc., 126 East Lincoln Avenue, P.O. Box 2000, Rahway, NJ, 07065, USA; email: xiaohan.hu@merck.com

**List of Supplementary Material**

**Supplementary Figure 1.** Patient Attrition

**Supplementary Figure 2.** Kaplan-Meier Analysis of Overall Survival (a) After 1 Year Post Complete Resection (Landmark Timepoint 1), (b) After 3 Years Post Complete Resection (Landmark Timepoint 2), (c) After 5 Years Post Complete Resection (Landmark Timepoint 3), Among Patients Without Recurrence Before the Landmark Timepoint

**Supplementary Figure 3.** Kaplan-Meier Analysis of Real-World Disease-Free Survival (a) After 1 Year Post Complete Resection (Landmark Timepoint 1), (b) After 3 Years Post Complete Resection (Landmark Timepoint 2), (c) After 5 Years Post Complete Resection (Landmark Timepoint 3), Among Patients Without Recurrence Before the Landmark Timepoint

**Supplementary Table 1.** Adjuvant Systemic Therapy Treatment Patterns for Patients with Completely Resected Stage IB-IIIA, Overall and by Disease Stage

**Supplementary Table 2.** Distribution of Type of Real-World Disease-Free Survival Events Among Patients Without Recurrence by Landmark Timepoints

**Supplementary Figure 1. Patient Attrition**

**
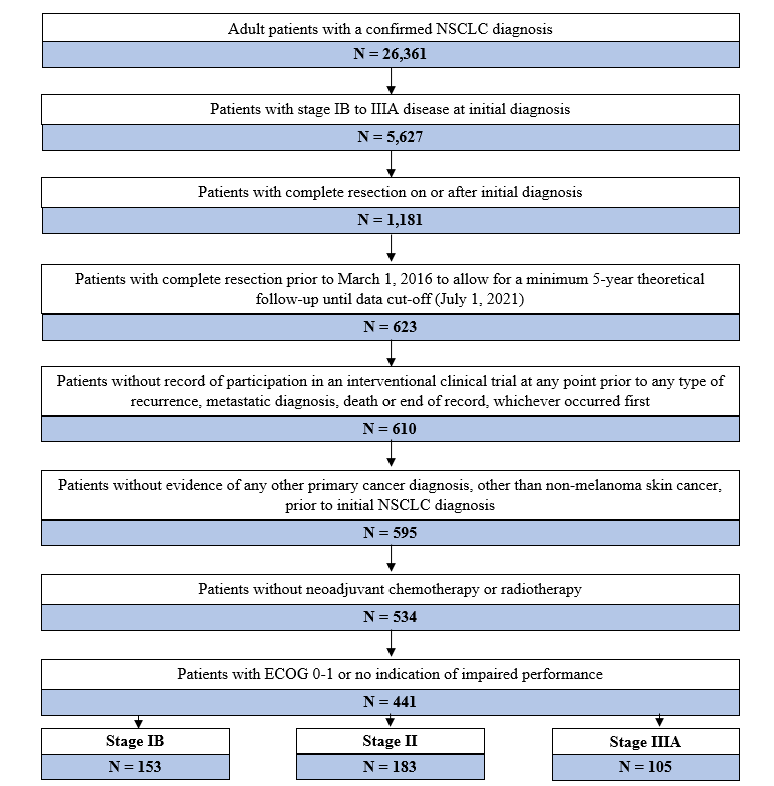
**

ECOG, Eastern Cooperative Oncology Group; NSCLC, non-small cell lung cancer

**Supplementary Figure 2.** Kaplan-Meier Analysis of Overall Survival (a) After 1 Year Post Complete Resection (Landmark Timepoint 1), (b) After 3 Years Post Complete Resection (Landmark Timepoint 2), (c) After 5 Years Post Complete Resection (Landmark Timepoint 3), Among Patients Without Recurrence Before the Landmark Timepoint


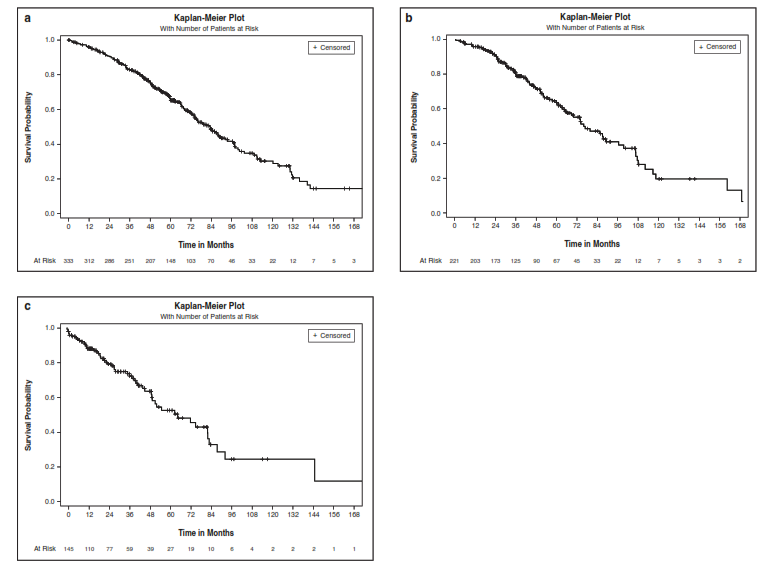


**Supplementary Figure 3.** Kaplan-Meier Analysis of Real-World Disease-Free Survival (a) After 1 Year Post Complete Resection (Landmark Timepoint 1), (b) After 3 Years Post Complete Resection (Landmark Timepoint 2), (c) After 5 Years Post Complete Resection (Landmark Timepoint 3), Among Patients Without Recurrence Before the Landmark Timepoint


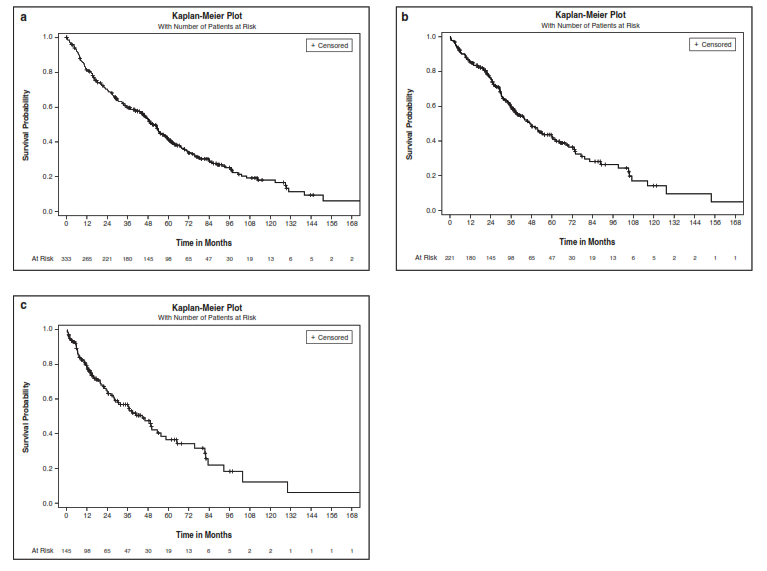


| **Supplementary Table 1. Adjuvant Systemic Therapy Treatment Patterns for Patients with Completely Resected Stage IB-IIIA, Overall and by Disease Stage** | | | | |
| --- | --- | --- | --- | --- |
| **Variable/Statistic** | **Overall**  **(N=441)** | **Patients with Stage IB (N=153)** | **Patients with Stage II (N=183)** | **Patients with Stage IIIA (N=105)** |
| Adjuvant Chemotherapy, n (%) |  |  |  |  |
| Patients receiving first adjuvant chemotherapy | 154 (34.9%) | 24 (15.7%) | 77 (42.1%) | 53 (50.5%) |
| Patients receiving second adjuvant chemotherapy | 26 (5.9%) | 5 (3.3%) | 11 (6.0%) | 10 (9.5%) |
| Patients receiving third adjuvant chemotherapy | 9 (2.0%) | 3 (2.0%) | 3 (1.6%) | 3 (2.9%) |

| **Supplementary Table 2. Distribution of Type of Real-World Disease-Free Survival Events Among Patients Without Recurrence by Landmark Timepoints** | | | |
| --- | --- | --- | --- |
|  | **1 Year Following Surgery** | **3 Years Following Surgery** | **5 Years Following Surgery** |
| No. of patients remaining disease-free at each landmark | 333 | 221 | 145 |
| No. of rwDFS events during available follow-up since each landmark | 219 | 123 | 73 |
| No. of recurrence events (%) | 157 (71.7%) | 77 (62.6%) | 39 (53.4%) |
| No. of death events without evidence of recurrence (%) | 62 (28.3%) | 46 (37.4%) | 34 (46.6%) |

rwDFS, real-world disease-free survival.

rwDFS was defined as the interval from the date of complete resection to the date of the first recurrence event (locoregional recurrence or distant recurrence, based on provider documentation), new diagnosis of other primary cancer other than non-melanoma skin cancer, or date of death, whichever occurred first.
